# Effect of Computational Method on Accumulated O_2_ Deficit

**DOI:** 10.3389/fspor.2022.772049

**Published:** 2022-03-07

**Authors:** Jon Ingulf Medbø, Boye Welde

**Affiliations:** ^1^Faculty for Teacher Education, Culture, and Sports, Institute of Language, Literature, Mathematics and Interpretation, Western Norway University of Applied Sciences, Bergen, Norway; ^2^School of Sport Sciences, University of Tromsø The Arctic University of Norway, Tromsø, Norway

**Keywords:** accumulated O_2_ deficit, anaerobic energy release, computation method, exercise economy, exercise efficiency, high-intensity exercise, measurement, O_2_ uptake

## Abstract

The aim of this study was to examine how relationships between exercise intensity and the rate of energy release established in different ways, affect the calculated O_2_ deficit accumulated during strenuous exercise. Aerobic energy release is readily measured by the O_2_ uptake, while anaerobic energy release is by definition independent of O_2_. The latter is not easily measured during strenuous exercise, but it can be estimated using the accumulated O_2_ deficit principle. We have calculated it using nine different approaches. Thirteen moderately trained persons (three women) volunteered to serve as subjects for cycle ergometry. Their maximal O_2_ uptake was 2.9 ± 0.6 mmol s^−1^ (*x̄* ± *s*; 3.9 ± 0.8 L_STPD_ min^−1^). Our reference method (M0) is based on measuring the steady state O_2_ uptake at the end of at least ten bouts of 10 min of exercise at constant intensity, varying between 30 and 40% of that corresponding to the maximal O_2_ uptake and up to a power >90% of the maximal O_2_ uptake, which is a rather time-consuming method. The outcomes of eight different simpler approaches have been compared with those of the reference method. The main result is that the accumulated O_2_ deficit calculated depends a great deal on the relationship used to calculate it. A protocol of stepwise increases in exercise intensity every 4 min appeared to work well. A gross efficiency method showed the poorest performance. Another important result is that at constant power the O_2_ uptake continued to increase beyond 4 min of exercise at all powers examined, also at powers well-below those corresponding to the lactate threshold. Finally, the O_2_ uptake during loadless pedaling was considerably higher than resting O_2_ uptake, and it appeared to follow a cubic function of the pedaling frequency. In conclusion, to obtain reliable values of the anaerobic energy release using the accumulated O_2_ deficit principle, reliable relationships between exercise intensity and O_2_ demand must be established.

## Introduction

Exercising muscles need energy (as ATP) that can be provided by aerobic and anaerobic processes. Aerobic energy release is readily measured by O_2_ uptake (e.g., Gastin, 2001). Anaerobic energy release is by definition independent of O_2_ and is not easily measured, for example in relation to strenuous exercise (e.g., Gastin, 1994; Medbø, 2010). Instead, the anaerobic contribution has been estimated by different computational methods. Medbø et al. (1988) proposed using accumulated O_2_ deficit as an indirect measure of the anaerobic energy release during high-intensity exercise. This principle seems to be the only reliable non-invasive measure available (Gastin, 1994). However, the original approach of Medbø et al. (1988) is time consuming and therefore not often used. Several short-cut approaches have been used, but with little validation (Medbø, 2010). In this invited paper we address how eight different (simpler) computational methods affect the calculated anaerobic energy supply in high-intensity activities. We limit our focus to computations involving the accumulated O_2_ deficit principle.

The main ideas of the accumulated O_2_ deficit principle are firstly that the rate of energy release (ATP turnover rate) appears to increase linearly with exercise intensity. Thus, if a relationship between exercise intensity and the rate of energy release is established reliably at moderate intensities, it can be extrapolated to higher intensities where anaerobic processes are important (Medbø et al., 1988). A second main idea is that since energy is provided by both aerobic and anaerobic processes, the anaerobic contribution can be taken as the difference between the total energy release and the aerobic part. The aerobic energy release is obtained from the O_2_ uptake measured during exercise.

The original approach proposed by Medbø et al. (1988) was to measure the O_2_ uptake at the end of 10 min of exercise at constant intensity, presumed to be reflect the total rate of energy release (called O_2_ demand). This must be performed 8–10 times or more at intensities ranging between 30 and 40% of the maximal O_2_ uptake and close to the maximum intensity that can be maintained for 10 min. It would thus require testing over several days.

Several simpler procedures may be proposed. It is widely held that the O_2_ uptake reaches a steady state value within 3 min of exercise (e.g., Whipp and Wasserman, 1986; Zoladz et al., 1998), at least for exercise below the lactate threshold (Hill and Lupton, 1923). If so, the duration of each exercise bout may be reduced from 10 to 4 min or less. Alternatively, exercise may be performed in small stepwise increases of exercise intensity (<30 W), for example every 4 min. The O_2_ uptake can be measured at the end of each step. If properly designed, that enables the establishment of the required relationship between exercise intensity and the rate of energy release in <1 h.

A linear relationship, here between exercise intensity (*x*; power for cycling) and O_2_ uptake (*y*), is described by the intercept and the slope. If the intercept is known, a reliable relationship can be obtained if the O_2_ demand at one or two intensities is known. The gross efficiency approach sets the intercept to zero. A net efficiency approach can be obtained by either measuring resting O_2_ uptake or O_2_ uptake at zero intensity exercise (for example loadless pedaling for cycle exercise) and using that value as the intercept. Medbø et al. (1988) found that while the slope varied considerably between subjects, the intercept did not. They consequently proposed using a common intercept for all subjects taken as the mean of all individual intercepts obtained.

The aim of this study was to examine how different relationships between exercise intensity and the rate of energy release (O_2_ demand) affect the calculated O_2_ deficit accumulated during strenuous exercise. We have therefore calculated the accumulated O_2_ deficit using nine different approaches. We have used the original approach proposed by Medbø et al. (1988) as the reference (called method 0, M0) and compared the outcome of eight other possible approaches (M1–M8) with those of the reference.

Our data are from cycling exercise. The principles addressed may be applied to other kinds of exercise too. In the discussion we address two further approaches to estimate the rate of energy release at high exercise intensities with a considerable anaerobic contribution that may be suited for treadmill exercise.

## Methods

### Overview

Moderately trained subjects repeatedly cycled at constant power for 10 min, and the O_2_ uptake was measured between 3 and 4 min of exercise and again at 8–10 min of exercise. The powers chosen ranged between loadless pedaling and close to the highest power that could be maintained for 10 min. Heart rate and blood lactate concentration were also measured. In further studies the exercise was repeated by using an exercise protocol of 4 min stepwise increases in power. The resting O_2_ uptake was also measured.

Relationships between power and O_2_ demand were established in nine different ways using data at different submaximal powers (below those corresponding to the maximal O_2_ uptake). The relationships obtained were extrapolated to powers corresponding to 100–250% of those corresponding to the maximal O_2_ uptake. The accumulated O_2_ deficit for (simulated) high-intensity exercise was calculated using the nine different relationships obtained.

### Subjects

Thirteen moderately trained volunteers served as subjects. Ten of them were students (three women, seven men), aged 24 ± 3 years (*x̄* ± *s*). In addition, three male staff members aged 32, 41, and 55 years also took part in some of the studies. The women were 1.71 ± 0.04 m tall and weighed 74 ± 7 kg. The men were 1.84 ± 0.05 m tall and weighed 82 ± 7 kg. The subjects' maximal O_2_ uptake was 2.9 ± 0.6 mmol s^−1^ (*x̄* ± *s*; 3.9 ± 0.8 L_STPD_ min^−1^).

The subjects were informed both orally and in writing about their rights as volunteers to leave the study at any stage without giving a reason for doing so, before giving their written consent. The study was non-invasive (except for sampling blood from a finger), included only adults capable of consent, and collected only anonymous data. It therefore did not require further ethical approval according to national regulations.

### Experiments

All exercise was performed as cycling on a modified Krogh-type cycle ergometer (Krogh, 1913) at a constant pedaling frequency, which in different experiments was set at between 0.5 Hz (30 rpm) and 2.0 Hz (120 rpm). Most experiments were carried out at 1.5 Hz (90 rpm) and again at 0.75 Hz (45 rpm). The power was varied by changing the braking force. The seat height was adjusted to each subject so that a straight leg reached the pedal without tilting the hip when the pedal was at the lowest position.

The total experimental set-up was quite comprehensive, and all subjects did not take part in all experiments. Twelve subjects performed repeated bouts (≈10–15 bouts for each subject) of 10 min continuous cycling at constant braking force at 1.5 Hz pedaling frequency. O_2_ uptake, heart rate and blood lactate concentration were measured at 3–4 min of exercise and again at 8–10 min. These tests took place over several days; each subject did 4–5 bouts of 10 min duration in each testing session, in increasing order of power. The power was varied between different bouts, ranging from loadless cycling (to allow comparison with resting O_2_ uptake) to close to a power that could barely be maintained for 10 min, thus on average 95% of that corresponding to maximal O_2_ uptake and well-above that corresponding to the lactate threshold. In total, these tests covered the range from loadless cycling to close to a power corresponding to maximal O_2_ uptake in steps no larger than 22 W. Nine subjects repeated these exercises when cycling at 0.75 Hz to allow for comparison of effects of different pedaling frequencies.

Nine subjects performed a protocol of stepwise increases in power every 4 min while cycling at 1.5 Hz. O_2_ uptake, heart rate and blood lactate concentration were measured near the end of each exercise step. The braking force was increased immediately after each 4 min period with no break or rest. The increase in power from one step to the next was 22 W for males and 11 W for females. The power was varied between loadless cycling and a power close to that corresponding to the maximal O_2_ uptake, thus being well above that corresponding to the lactate threshold. Eight subjects repeated this protocol when cycling at 0.75 Hz.

O_2_ uptake was measured at rest for ten subjects while the subject sat quietly on the cycle ergometer for 10 min. Nine subjects also cycled at zero load at a pedaling frequency of 0.5 Hz (30 rpm) to allow comparison with the O_2_ uptake at rest.

For eleven subjects the maximal O_2_ uptake was established by the leveling-off criterion of Taylor et al. (1955), modified for ergometer cycling as described by Hermansen (1974). More specifically, the subjects cycled at a high power for 3 min, and the O_2_ uptake was measured during the last 30–40 s. The test was repeated two to five times for new 3 min bouts at higher powers with a new measurement of the O_2_ uptake at the end until the O_2_ uptake did not increase for a further increase in power. The highest value obtained was taken as the subject's maximal value. It appeared that the highest O_2_ uptake measured during the repeated 10 min bouts or the 4 min stepwise increasing power for these subjects was on average 95% of the maximum value established by the leveling-off criterion. Two subjects did not carry out the repeated 3 min bouts. For these two subjects the highest measured O_2_ uptake measured during the tests was used to indicate their maximum value.

### Equipment

All cycling was done on a Krogh-type cycle ergometer (Krogh, 1913). The ergometer has an instrument recording the pedaling frequency by optical recordings from the sprocket crank wheel. A possible deviation from the preset frequency is shown on an analog instrument. This enables the subjects to keep the pedaling rate constant.

Oxygen uptake was measured by the Douglas bag technique (Douglas, 1911). Expired air was collected in Douglas bags while the sampling time was recorded. After collection the closed bag was transferred to a bench and emptied, while the sampled volume was measured with an S430-A ventilation measuring system with a K520–C521 flow transducer (Applied Electrochemistry, Pittsburgh, PA, USA) while the air temperature was measured simultaneously by a digital thermometer. During the emptying a small sample of expired air was extracted to measure fractions of O_2_ and CO_2_ with instruments of Applied Electrochemistry; O_2:_ an S 3A/I analyzer with an N-22M zirconium oxide-type O_2_ sensor; CO_2_: a CD-3A analyzer with a P-61B infrared-type CO_2_ sensor). The instruments were calibrated by high-quality gases of known concentrations, and by a 7 L syringe (series 4900, Hans Rudolph, Kansas City, MO, USA). Heart rate was measured by a PE 3000 heart rate recorder (Polar Electro OY, Kempele, Finland). Blood lactate concentration was measured from blood taken from a cleaned finger by the LT-1710 Lactate Pro™ analyzer (Arkray Factory Inc., KDK Corporation, Shiga, Japan). A separate examination has shown that this instrument measures blood lactate concentration quite accurately, with a random error of 0.5 mmol L^−1^ or less (Medbø et al., 2000). The lactate threshold was taken as the exercise intensity corresponding to a blood lactate concentration of 4.0 mmol L^−1^, as established by a linear interpolation if necessary. This is a simple, straightforward criterion that has been shown to provide a value close to the maximal lactate steady state criterion (Mamen et al., 2011).

### Computational Methods Examined

We examined eight different methods for establishing the relationship between power and O_2_ demand as alternatives to the one proposed by Medbø et al. (1988; called M0 here). For all models (described below) it is assumed that O_2_ demand increases linearly with exercise intensity that for cycling is taken as the power. The first four methods tested (M1–M4 below) use around ten pairs of values for each subject to estimate both the slope and the intercept (not M2) of the relationships in question.

**Figure 1:**
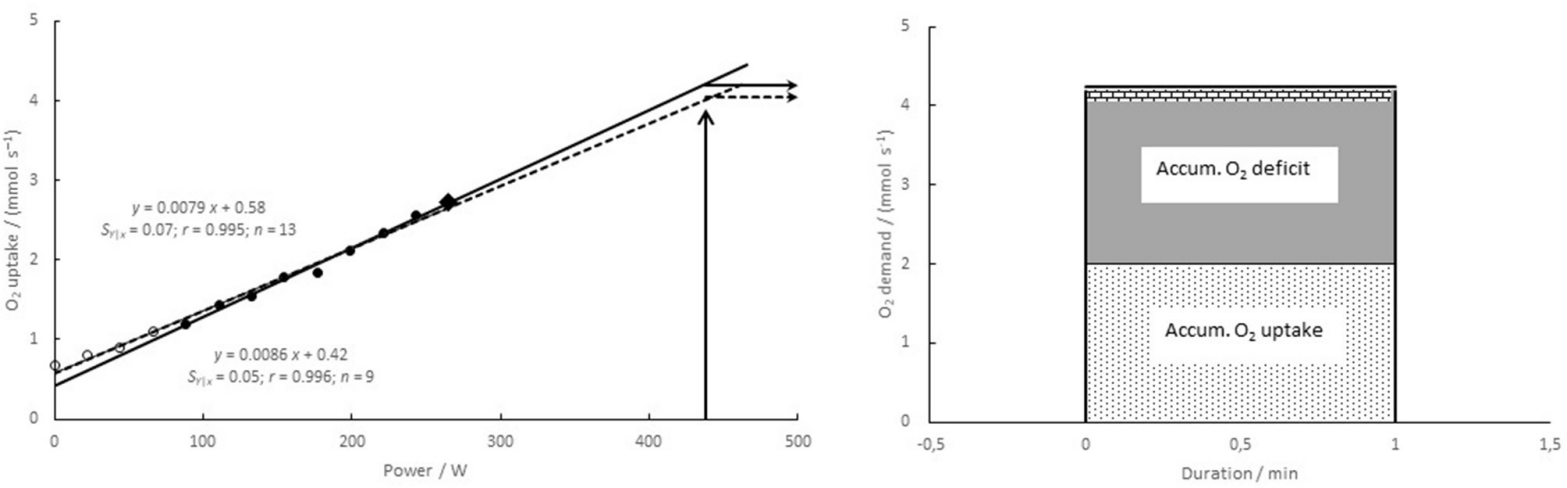
**(Left)** relationship between power and O_2_ demand for a typical subject (no. 8). O_2_ uptake was measured at 8–10 min of exercise at nine different constant powers between 88 and 265 W (filled symbols), and a linear regression was calculated based on those data. The highest value (filled diamond) was used in methods M5–M8 (see Figure 2 below). If in addition four measurements at lower powers are included (open symbols), a different relationship with a higher value of the intercept and a lower slope is obtained (dashed line, called method 4). **(Right)** calculations of the accumulated O_2_ deficit. O_2_ uptake was measured during 1 min exercise to exhaustion (here taken from a previous study), giving the accumulated O_2_ uptake (light dotted area). The accumulated O_2_ demand is taken as the O_2_ demand times the duration, reflecting the total energy release in units of oxygen. The accumulated O_2_ deficit (dark gray area + hatched area at the top) is taken as the accumulated O_2_ demand minus the accumulated O_2_ uptake. If the O_2_ demand is estimated to be lower (here by method 4), the estimated accumulated O_2_ deficit becomes lower (hatched area at the top not included).

M0 Method 0, proposed by Medbø et al. (1988), was used as the reference method. It is based on repeated measurements of the steady state O_2_ uptake for at least 8–10 different bouts at constant power of 10 min duration. The powers used varied between 30 and 40% and >90% of that corresponding to the maximal O_2_ uptake. There are non-linear effects at low powers (see Figure 1 for an example), and measurements below the non-linear threshold have not been included. The regression coefficients were found by ordinary least square regression.
M1 Method 1 uses data gathered as for method 0, but the O_2_ uptake was measured between 3 and 4 min of exercise during a 10 min bout. The only difference between methods 0 and 1 is the duration of cycling before a measurement is taken.
M2 Method 2 uses the same data as method 1, but in addition the y-intercept is a forced value (the mean of the intercepts of method 1; 0.50 mmol s^−1^ for the present data). The only difference between methods 1 and 2 is the forced y-intercept of method 2.
M3 In method 3 exercise starts at a rather low power, <100 W. Between 3 and 4 min of exercise, O_2_ uptake was measured. Immediately after the measurement the intensity was raised by a small step (11–22 W, depending on the subject's physical level; no break or rest between steps), and a new 4 min cycle was repeated. This procedure was repeated at least ten times, giving more than ten paired measurements of the O_2_ uptake vs. power in <1 h. The regression coefficients were determined using least square regression.
M4 As shown in Figure 1, there are non-linear effects at low powers. Including measurements at low power will affect the calculated relationships between power and O_2_ uptake. Method 4 includes all measurements, even those at loadless pedaling. The regression coefficients were calculated by least square regression.

**Figure 2:**
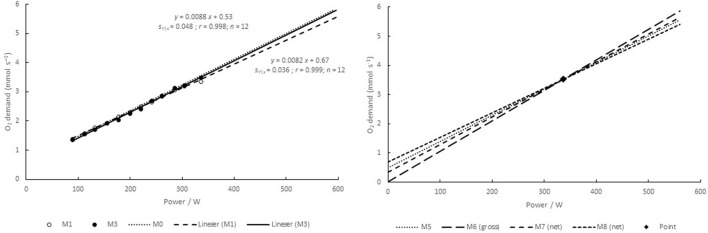
Relationship between power and O_2_ demand for a typical subject (no. 9), and the effects of extrapolations to high powers. **(Left)** relationships established by methods 1 and 3 (M1 and M3). The relationship of M2 is very close to that of M1, and it is therefore not included. The relationship of the reference method (M0) is shown by the dotted line. A corresponding relationship for M4 is shown in Figure 1. **(Right)** relationships established by methods 5–8 (M5–M8) using fixed intercepts and one measurement at around 90% of the maximal O_2_ uptake (point). The line of M5 is very close to that of M0, which is therefore not included.

The last four methods use a fixed intercept and only one or two individual measurements. The slope is found as follows: The O_2_ uptake is measured near the end of a 10 min bout at a power corresponding to around 90% of the maximal O_2_ uptake. A linear relationship is taken from the intercept (presumed O_2_ uptake at zero power) through the measured O_2_ uptake and the corresponding power (Figure 2).

M5 For method 5 the mean of the individual intercepts (see methods 0 and 2) was calculated and used as a common intercept for all subjects. O_2_ uptake is measured at a power corresponding to ≥90% of maximal O_2_ uptake (e.g., diamond in Figure 1), and a linear relationship is taken from the intercept through the measured value.
M6 Method 6, the gross efficiency method, sets the intercept to zero. The O_2_ uptake at a high power (≥90% of maximal O_2_ uptake) is measured, and a linear relationship is taken from the zero intercept through this measured value.
M7 Method 7 is a net efficiency method. The O_2_ uptake is first measured at rest, giving the intercept, and then at a power corresponding to ≥90% of the maximal O_2_ uptake. A linear relationship is taken from the intercept through this latter measured value.
M8 Method 8 is also a net efficiency method. The O_2_ uptake is first measured during loadless cycling, giving the intercept, and then at a power corresponding to ≥90% of the maximal O_2_ uptake. A linear relationship is taken from the intercept through this latter measured value.

Method 0 has been used in some studies (Medbø et al., 1988; Medbø and Tabata, 1989, 1993; Tabata et al., 1996, 1997; Buck and McNaughton, 1999). Method 1 has been used by Green and Dawson (1996) and further examined by Buck and McNaughton (1999). Method 2 has been recommended by Noordhof et al. (2010, 2013). Method 3 has been used by Duffield et al. (2007). Bosquet et al. (2007a,b) and Riojas et al. (2020) used a similar protocol with 2-min steps, thus increasing the power well before the O_2_ uptake reaches a (near) steady state. Method 3 has also been recommended by Medbø et al. (1988) and by Medbø (2010). While Medbø et al. (1988) warned against possible flaws when using method 4, they recommended method 5 as an alternative to the proposed reference method. Method 5 has later been used in many studies (Scott et al., 1991; Ramsbottom et al., 1994, 1997, 2001; Maxwell and Nimmo, 1996; Doherty, 1998; Wadley and le Rossignol, 1998; Doherty et al., 2000, 2002; Russell et al., 2000; Bickham et al., 2002; Nevill et al., 2008). The gross efficiency principle (method 6) has been recommended by many (e.g., Ettema and Lorås, 2009; Noordhof et al., 2013) and more recently used for cross-country skiing (Andersson and McGawley, 2018). The two net efficiency methods (no. 7 and 8) do not seem to have been greatly used.

### Calculations and Modeling

For high intensity exercise, anaerobic energy release can be estimated by the accumulated O_2_ deficit, assuming that the O_2_ uptake measured at the end of the 10 min bouts described above is equal to the total rate of energy release in those exercises. The established linear relationship between exercise intensity and the rate of energy release is also assumed to hold for higher intensities where there is a considerable anaerobic contribution. The O_2_ uptake is measured throughout the whole high intensity exercise, giving the accumulated O_2_ uptake as the aerobic contribution. The anaerobic part, called the accumulated O_2_ deficit, is taken as the difference between the total energy release and the measured aerobic part (Figure 1 above); see Medbø et al. (1988 or 2010) for further details. This means that different relationships between exercise intensity and energy release will give different values for the accumulated O_2_ deficit calculated, as shown in Figure 1.

Exercise at very high intensity leads to exhaustion in a few seconds. Exercise may be maintained for a longer duration if the load is lighter. In our modeling we assume exercise at constant intensity to exhaustion. The present study does not include experiments at powers above that corresponding to the maximal O_2_ uptake. To allow extrapolations to supramaximal intensities we have used data from our former studies (Table 1). More specifically, the data of Medbø and Tabata (1989) and Medbø et al. (1999) suggest that to cause exhaustion in 10 and 30 s typical subjects must cycle at a power corresponding to ≈250 and 200%, respectively, of their maximal O_2_ uptake. The data of Medbø and Tabata (1989) suggest that the average subject can exercise at an intensity ≈150 and 120% of that corresponding to the maximal O_2_ uptake, for 1 and 2 min, respectively, before exhaustion is reached. By an extrapolation beyond their measurements, their data also suggest that exercise at a power corresponding to 100% of the maximal O_2_ uptake will exhaust the subject in about 3.6 min (exactly 217 s). This latter estimate is at variance with experience from laboratory experiments. We have nevertheless used this value as an estimate for further modeling, which we address again in the discussion.

**Table 1:** Assumptions for the calculations and modeling.

**Exercise intensity/** **(percent of max. O_2_ uptake)**	**Assumed time to exhaustion**	**Accumulated O_2_ uptake/** **(percent of total energy release)**	**O_**2**_ demand/** **(mmol s^**−1**^)**	**Estimated power/W**
100	3.62 min (217 s)	75	2.9	274
120	2 min (120 s)	63	3.5	343
150	1 min (60 s)	47	4.4	438
200	30 s	30	5.8	603
250	10 s	10	7.3	768

*It is assumed that the subject exercises at a constant, preset power to exhaustion. Data for exercise duration at different supramaximal intensities are taken from Medbø and Tabata (1989) and Medbø et al. (1999) The accumulated O_2_ uptake as a proportion of the total energy release is estimated from a model by Medbø (2010). The O_2_ demand for each exercise is taken from the average subject's maximal O_2_ uptake and the exercise intensity relative to that maximal. The corresponding power has been calculated from the O_2_ demand using the mean of the regression parameters of the subjects. See the main text for further details*.

Medbø (2010) pooled data from 30 different studies on the relative contribution of O_2_ taken up during exercise to the total energy release. Exercise duration ranged from about 10 s to more than 5 min, and all exercises were performed to exhaustion. The model produced enables estimation of the accumulated O_2_ uptake as a proportion of the total energy release as a function of the time to exhaustion (see Figure 8 in Medbø, 2010), and these estimates are summarized in Table 1. Consequently, when estimating the total rate of energy release by our reference method, the accumulated O_2_ uptake is estimated to be 75% of the total for the 3.6 min exercise to exhaustion, and 10% of the total for the 10 s sprint at 250% of the maximal O_2_ uptake (Table 1).

To apply these values to our data for further calculations, we used average values of our 13 subjects, for example the average maximal O_2_ uptake of 2.9 mmol s^−1^ (3.9 L min^−1^). Therefore, an exercise intensity of 120 and 250% of the maximal O_2_ uptake corresponds to an O_2_ demand of 3.5 and 7.3 mmol s^−1^, respectively.

The total rate of energy release (*Y*) is assumed to be related to the power (*P*) by the linear relationship


$$\begin{array}{l} Y=\;a+b\;P \end{array}$$

where *a* and *b* are, respectively, the intercept and slope of the linear relationships. This equation is readily solved for the power, giving


$$\begin{array}{l} P=\;\frac{Y-a}{b} \end{array}$$

The power for each exercise model in Table 1 was calculated using the regression coefficients obtained for the reference method (M0 above). Thus, exercise at 120 and 250%, for example, corresponds to a power of 343 and 768 W, respectively, for the average subject (Table 1).

For linear relationships obtained by different protocols we have assumed the same power, time to exhaustion, and accumulated O_2_ uptake. The estimated O_2_ demand and thus the (estimated) total energy release will differ depending on the linear parameters used, as will the accumulated O_2_ deficit calculated by the different models.

All calculations were performed on a spreadsheet (Excel version 2021).

### Statistics

Results are summarized as mean ± $s\left(\bar{x}\right)$ (standard error of the mean). Possible changes were examined by Gosset's (“Student's”) one-sample *t*-test for matched-pair measurements on the same subject, and further by a two-way analysis of variance (IBM SPSS version 27), using Dunnett's *post-hoc* test (M0 as the reference). Linear regressions were calculated using the built-in regression tools in the spreadsheet (in the package for data analysis). Non-linear curve fits (cubic and square) were calculated by using the option of polynomial regression in the plotting menus.

Not all subjects carried out all experiments. When some data are missing from a subject, the statistical analysis used automatically excludes all data for that subject for the test in question. The effect is a somewhat lower test power, while no systematic effect is introduced.

## Results

### O_2_ Uptake vs. Exercise Protocol

#### 1.5 Hz Cycling

O_2_ uptake was measured between 3 and 4 min of exercise and again between 8 and 10 min of cycling for 10 min at constant power (between 70 and ≈300 W) at 1.50 Hz. There was a small but statistically significant increase in the O_2_ uptake of 0.044 ± 0.005 mmol s^−1^ during the last 6 min of exercise (*n* = 155 paired measurements on 12 subjects; +2.7%; *P* < 0.001). For measurements below the individual lactate threshold the increase was 0.026 ± 0.004 mmol s^−1^ (*n* = 126 measurements; +1.8%; *P* < 0.001), while for measurements at or above the threshold the increase was 0.113 ± 0.012 mmol s^−1^ (*n* = 33 values; +4.9%, *P* < 0.001). The blood lactate concentration measured just after the highest power maintained for 10 min was 8.9 ± 1.8 mmol L^−1^ (*x̄* ± *s*).

Nine subjects performed cycling at stepwise increasing power every 4 min. O_2_ uptake was measured during 3–4 min of exercise at each step. O_2_ uptake was 0.015 ± 0.007 mmol s^−1^ higher during 4 min stepwise increasing power than after 4 min of cycling with rest before a new exercise (*P* = 0.03). O_2_ uptake was 0.026 ± 0.007 mmol s^−1^ lower during 4 min stepwise increasing power than after 10 min of cycling at the same power with rest before the exercise (*P* < 0.001).

#### 0.75 Hz Cycling

Nine subjects cycled at 0.75 Hz for 10 min at different constant powers where the O_2_ uptake was measured at 3–4 and 8–10 min of cycling. The findings were similar to those at 1.50 Hz. In brief, the O_2_ uptake increased with time both for cycling below (+2.1%; *n* = 109 paired comparisons; *P* < 0.001) and at or above the lactate threshold (+5.1%; *n* = 19; *P* < 0.001).

Eight of these subjects performed the protocol of 4 min stepwise increasing power at 0.75 Hz frequency. O_2_ uptake was measured during 3–4 min of exercise at each step and compared with the values obtained at the same power after 4 and 10 min of cycling with a rest before a new exercise bout. For 106 paired comparisons the values of the two 4 min protocols did not differ (−0.002 ± 0.007 mmol s^−1^ for the stepwise protocol vs. after 4 min of 10 min bouts). The O_2_ uptake was 0.033 ± 0.006 mmol s^−1^ lower during 4 min stepwise increasing power than after 10 min of cycling with rest before each exercise (*P* < 0.001).

### O_2_ Uptake at Low Powers

O_2_ uptake was measured during loadless pedaling and for increasing powers up to close to the maximum that could be maintained for 10 min. For powers below about 75 W (≈1 W kg^−1^ body mass) the measured O_2_ uptake was somewhat higher than that expected based on linear extrapolation of relationships established at higher powers (see Figure 1 above for a typical example). Consequently, measurements at powers <75 W were not included (except for method 4). For cycling at 0.75 Hz the non-linear effect was less pronounced. At that frequency the lower limit was set to 30 W. Including measurements below the proposed lower limit resulted in relationships with a higher intercept but a lower slope, as addressed further below (see results for method 4).

### Intercept and O_2_ Uptake vs. Pedaling Frequency

**Figure 3:**
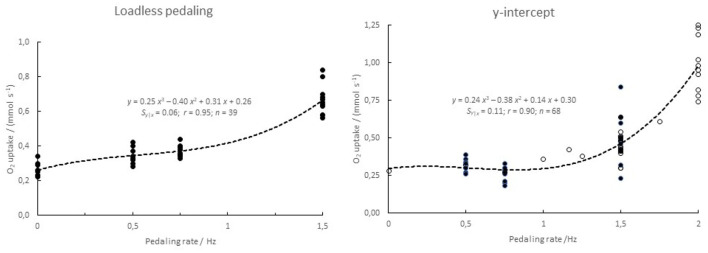
O_2_ uptake as a function of pedaling frequency. **(Left)** loadless pedaling of *n* = 8–11 subjects. **(Right)** y-intercept of linear regressions of steady-state O_2_ uptake on power. The data for the latter curve are taken from this study (0.5, 0.75 and 1.5 Hz; *n* = 8, 10, and 12, respectively, filled symbols) and from Medbø and Tabata (1989) and Medbø et al. (1999; open symbols).

O_2_ uptake was measured at rest while the subject sat still on the cycle ergometer and further during loadless pedaling at 0.5, 0.75, and 1.5 Hz cycling frequency. The data obtained are compatible with a cubic (also a square) relationship between the frequency and the O_2_ uptake (Figure 3, left panel). The y-intercept was established at different pedaling rates. The data are compatible with the idea that the *y*-intercepts follow a cubic (also square, not shown) relationship with the pedaling frequency (Figure 3, right panel).

### Effect of Relationship Between Power and O_2_ Uptake on Estimated Accumulated O_2_ Deficit

Both the intercepts and the slopes differed systematically between the different methods (*P* < 0.001). More specifically, Dunnett's *post-hoc* test revealed different intercepts of methods M4, M6, M7, and M8 vs. M0. There was no significant difference in the intercepts of M1 or M3 vs. that of M0 (and obviously no difference for M2 and M5 using mean values as intercepts). Likewise, the slopes of M1, M4, M6, M7, and M8 differed significantly from that of M0, while the slopes of M2, M3, and M5 did not. Consequently, the relationships also differed systematically when extrapolated to intensities above that corresponding to the maximal O_2_ uptake, which is shown in Figures 1, 2 above.

The accumulated O_2_ deficit for five different exercise intensities to exhaustion was calculated by the nine different methods as explained above. The values differ significantly between the models used (*P* < 0.001) as well as between subjects and exercise durations (*P* < 0.001). The results are summarized in Table 2.

**Table 2:** Intercept and slope of nine different approaches examined, and the accumulated O_2_ deficit calculated for each approach for five different exercise conditions.

		**Intercept**	**Slope**	**Accumulated O_2_ deficit/(mmol/kg body mass)**				
		**mmol O_**2**_ ·s^**−1**^**	**μmol O_**2**_ ·J^**−1**^**	**Exercise model (time to exhaustion, and intensity relative to max. O_2_ uptake)**				
**No**.				**10 s, 250%**	**30 s, 200%**	**60 s, 150%**	**2 min, 120%**	**3.6 min, 100%**
	**Methods based on many individual measurements**							
M0	10 min bouts (reference)	0.48	8.77	0.82	1.52	1.73	1.95	1.97
M1	4 min bouts with rest	0.50	8.38	0.77	1.42	1.58	1.71	1.64
M2	4 min bouts with rest, common y-intercept	0.50	8.51	0.78	1.45	1.62	1.78	1.74
M3	4 min stepwise increasing power	0.45	9.00	0.82	1.53	1.73	1.94	1.94
M4	10 min bouts, including low powers	0.60	8.24	0.77	1.43	1.63	1.83	1.86
	**Methods assuming a fixed intercept and few measurements**							
M5	Fixed, measured intercept + 10 min bout(s) at high(est) power(s)	0.48	8.71	0.80	1.48	1.67	1.86	1.84
M6	Gross efficiency	0	10.80	0.94	1.77	1.99	2.20	2.07
M7	Net efficiency from resting O_2_ uptake	0.26	9.66	0.86	1.61	1.81	2.01	1.93
M8	Net efficiency from O_2_ uptake at loadless cycling	0.66	7.95	0.75	1.38	1.56	1.73	1.75
	Average *s*($\bar{x}$)	0.04	0.22	0.03	0.06	0.08	0.10	0.14

*Details of the nine different models are given in the methods. The values for the slopes and intercepts (except for M6) are the mean of the individual values. Average s(x̄) denotes the standard error of the mean, averaged across the nine methods*.

### Methods Based on Many Individual Data

**Figure 4:**
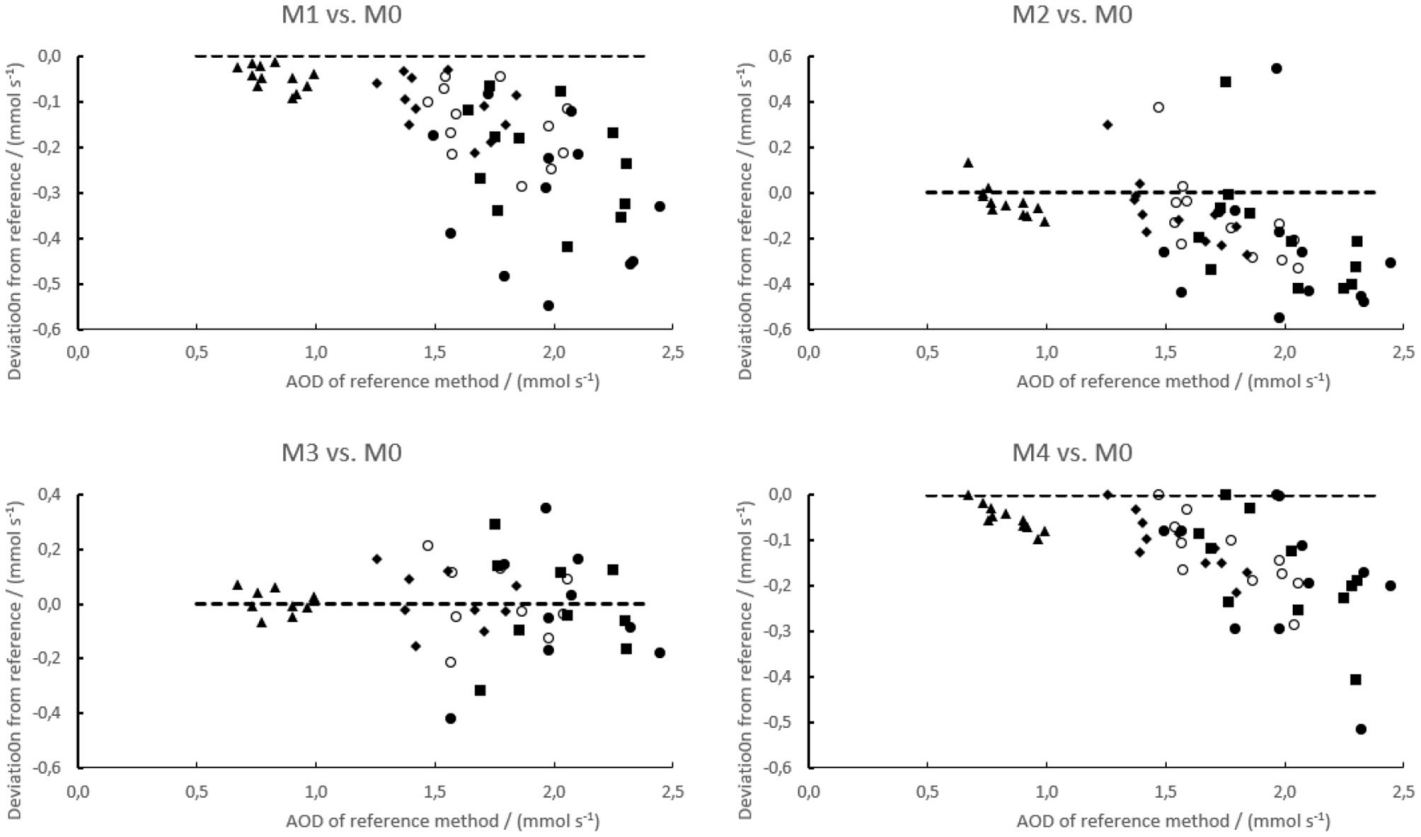
Residual plots of the accumulated O_2_ deficit calculated for four examined test methods (M1–M4) vs. the reference method (M0). The residuals have been taken as the value of the test method less that of the reference method. The data are simulated for exercise to exhaustion within 10 s (250% of max. O_2_ uptake, ▴), 30 s (200% of max. O_2_ uptake, ♦), 1 min (150% of max. O_2_ uptake, ◯), 2 min (120% of max. O_2_ uptake, ■), and 3.6 min (100% of max. O_2_ uptake, ∙) as explained in the methods. The dashed line is the zero line, indicating no difference. Note that the *y*-axes are not scaled equally.

The results obtained when using O_2_ uptakes measured after 3–4 min of exercise instead of 10 min bouts (methods 1 and 2) are systematically lower than those of the reference method (*P* < 0.001), in particular for the longest exercise durations where the values were around 15% lower than those of the reference method (Figure 4, upper left panel). The main reason for the lower estimates is the lower slope coefficient. Using a fixed, common y-intercept (method 2) apparently reduced the difference to the values of the reference method (Table 2). The main reason is a number of outliers from one subject (see Figure 4, upper right panel).

The results of the 4 min stepwise increases in power (method 3) are on average very close to those of the reference method for all five exercise combinations examined. Method 4 examined the effect of including all measurements at the end of 10 min bouts for calculating the regression parameters, even those at low powers where non-linear effects were seen. In this case the calculated values were systematically lower (around 6%) than those obtained by the reference method (*P* < 0.001).

### Methods Assuming a Fixed, Common Intercept and Few Individual Values

**Figure 5:**
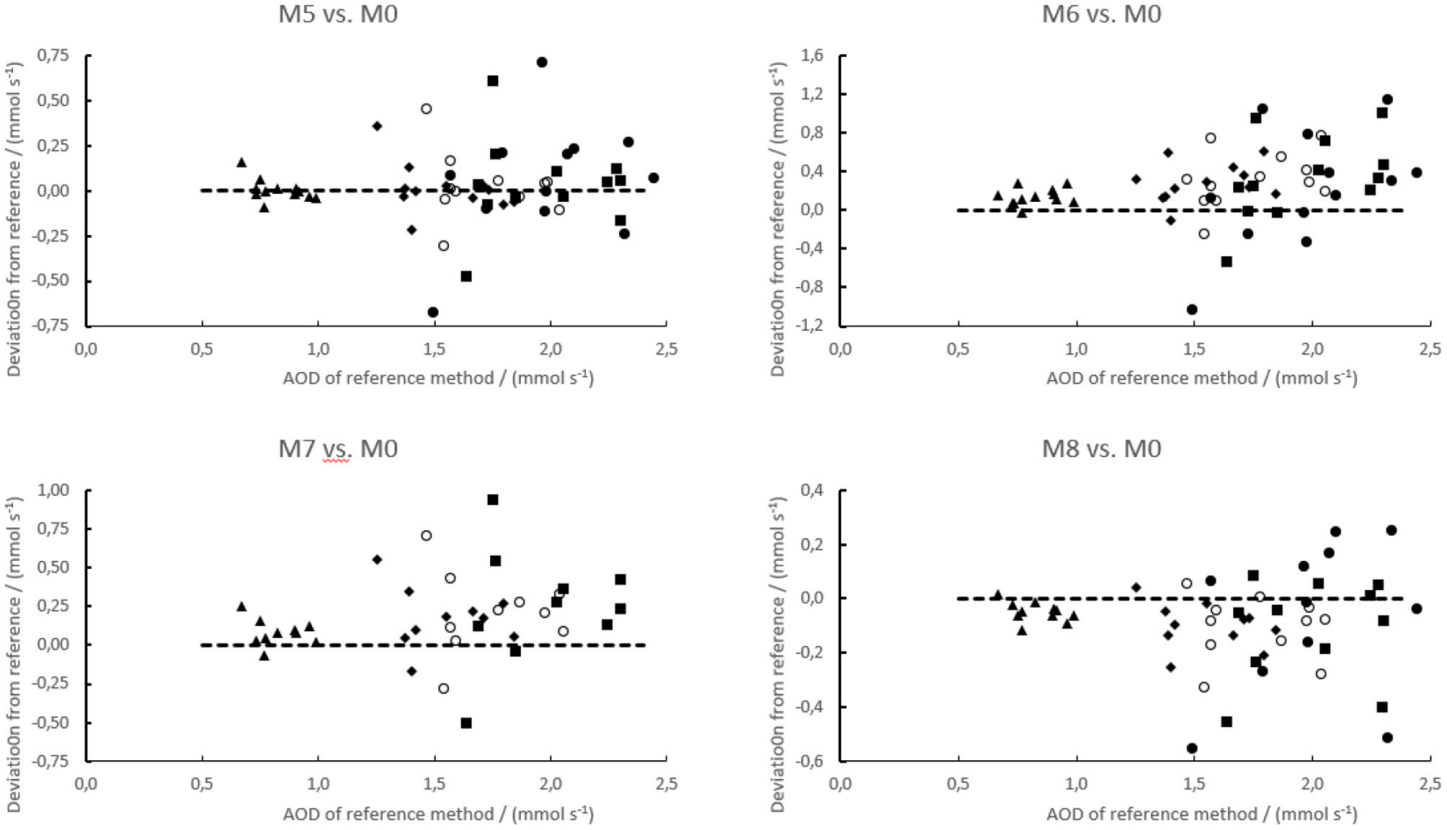
Residual plot of the accumulated O_2_ deficit calculated for four examined test methods (M5–M8) vs. the reference method (M0). The residuals have been taken as the value of the test method less that of the reference method. The data are simulated for exercise to exhaustion within 10 s (250% of max. O_2_ uptake, ▴), 30 s (200% of max. O_2_ uptake, ♦), 1 min (150% of max. O_2_ uptake, ◯), 2 min (120% of max. O_2_ uptake, ■), and 3.6 min (100% of max. O_2_ uptake, ∙) as explained in the methods. The dashed line is the zero line, indicating no difference. Note that the *y*-axes are not scaled equally.

Method 5 is based on a common intercept taken as the mean of all subjects. The slope was taken assuming a linear relationship from that intercept through the point corresponding to the highest power that was maintained for 10 min, and the corresponding O_2_ uptake. The slope of this approach was similar to that of the reference method, and consequently the accumulated O_2_ deficit calculated by this method was on average only ≈1% larger than the values of the reference method (Figure 5).

The gross efficiency method (no. 6) has a zero intercept and a slope that was nearly 13% higher than that of the reference method. This method gave values ≥15% larger than those of the reference method (*P* < 0.001). This method also showed the largest between-subjects variability.

Two versions of the net efficiency method were examined. For the one assuming a linear relationship from resting O_2_ uptake at zero load (no. 7), the intercept was lower and the slope was 9% higher than that of the reference method. The accumulated O_2_ deficit calculated by this method was on average ≈5% higher than the corresponding values of the reference method (*P* < 0.001). For method number 8 the net efficiency was calculated using the O_2_ uptake measured during loadless pedaling as the intercept. The slope of this method was 8% lower than that of the reference method, and consequently the accumulated O_2_ deficit calculated by this method was on average ≈10% lower than the corresponding values obtained by the reference method (*P* < 0.001).

### Variations Between Subjects

Figures 4, 5 show that there was considerable variability between subjects in performing the tested methods vs. the reference method. Both the average bias (addressed above) and the between-subjects variations differed systematically between the methods (Table 3). The between-subjects variations were least for methods 1 and 4, also quite small for method 3 that appeared unbiased, and largest for method 6 (gross efficiency). Moreover, the methods using a fixed y-intercept (M2 and M5–M8) showed in general larger variability than those establishing the intercept individually (M1, M3, and M4). In particular, methods 1 and 2 used the same data except that for method 2 a common y-intercept was used. Data of one outlying subject appeared to reduce the bias vs. the reference method but increase the between-subjects variation for method 2 compared with method 1.

**Table 3:** Between-subjects variations in differences in accumulated O_2_ deficit calculated by the eight methods examined vs. the reference method.

**Method**	**10 s, 250%**	**30 s, 200%**	**1 min, 150%**	**2 min, 121%**	**3.6 min, 100%**
M1	0.03	0.06	0.08	0.11	0.15
M2	0.07	0.15	0.19	0.25	0.29
M3	0.05	0.11	0.14	0.18	0.23
M4	0.03	0.06	0.08	0.12	0.15
M5	0.06	0.14	0.18	0.25	0.33
M6	0.09	0.20	0.28	0.43	0.61
M7	0.09	0.19	0.26	0.38	0.50
M8	0.04	0.08	0.12	0.18	0.28

*M1–M8 refer to methods 1–8. The values are the standard deviations for 9–12 subjects and expressed in mmol O_2_ s^−1^*.

## Discussion

The main result of this study is that the calculated value of the accumulated O_2_ deficit depends a great deal on the relationship used to calculate the value. The relationship changes if O_2_ at low exercise intensity is included. Further, the relationships are also influenced by the pedaling frequency.

### Methodological Considerations

We used data from repeated bouts of 10 min duration. The reason for that choice is that the O_2_ uptake did not reach a steady state within 3 min of exercise, even if the exercise intensity was well below that corresponding to the lactate threshold. Data from our previous study suggest that there is little change beyond 10 min of exercise (Medbø and Tabata, 1993). It is widely held that below the lactate threshold the O_2_ uptake reaches a steady state value within 3 min of exercise at constant intensity (e.g., Whipp and Wasserman, 1986; Zoladz et al., 1998). Our data, based on more than one hundred paired comparisons, suggest that that view needs to be revised.

Method 0, based on repeated bouts of 10 min duration, is our reference method. It has been argued that because of the slow component in the O_2_ uptake by time at exercise intensities above the lactate threshold, the reference method will overestimate the anaerobic energy release. Consequently, methods 1 or 2 might be more reliable (e.g., Noordhof et al., 2010, 2013). However, Tabata et al. (1997) found good agreement between the accumulated O_2_ deficit obtained during 4 min intermittent cycling at 170% of the maximal O_2_ uptake, and the maximal accumulated O_2_ deficit established by the reference method, when cycling continuously at ≈120% of the maximal O_2_ uptake to exhaustion. That would not be the case if the two quantities had been calculated using methods 1 or 2. Likewise, data by Buck and McNaughton (1999) suggest that the accumulated O_2_ deficit calculated by our reference method provides reliable data, while method 1 underestimates the value considerably.

We have calculated the accumulated O_2_ deficit from modeled data with no direct measurements of high-intensity exercise. One consequence of the modeling was that exercise at an intensity of no more than 100% of that corresponding to the maximal O_2_ uptake apparently leads to exhaustion in 217 s (3.6 min). This is clearly in conflict with common experience. However, we use these data only for comparisons of the outcome of different calculations. The effect of a misestimate will affect the different computational methods in a similar way, and thus not affect the outcome of the comparisons. These considerations suggest on the other hand that better models may be needed in future studies.

Our data in Figure 3 suggest that the O_2_ uptake during loadless cycling and the intercepts of the relationships of O_2_ uptake vs. power can be described by a cubic function of the pedaling frequency. That is compatible with a response in proportion to the power for accelerating the legs (see e.g., Formenti et al., 2015). However, while the leg during (loaded) cycling is accelerated during the first part of a push downwards, thus gaining kinetic energy, it is decelerated during the later part of the push, transferring its kinetic energy to the ergometer, as also addressed by Ettema and Lorås (2009). Corresponding considerations hold for kinetic energy added to the leg during a (passive) lift during the upstroke. These considerations suggest that other factors than accelerations and decelerations of the legs may cause the excess O_2_ uptake seen at high pedaling rates. There may for example be compensatory use of muscles in the upper body, muscle work that may not be regained as mechanical work on the ergometer. It is not known to what extent the observed cubic relationships really reflect a causal or mechanistic relationship. In line with that, second-order polynomials fit the data almost as well as the cubic ones used (not shown). The data nevertheless support the idea that at high speeds effects related to accelerations and decelerations may be important.

We measured O_2_ uptake with the Douglas bag technique and measured volume and fractions of O_2_ and CO_2_ in inspired and expired air using high-quality instruments. This allowed us full control of the outcome, including how the data (e.g., O_2_ uptake) are calculated from the measured values. The performance of automatic systems may on the other hand be influenced by built-in corrections (Medbø, 2002, 2010).

### Evaluation of the Different Calculation Models

#### Methods Using Many Individual Values

Method 1, repeated bouts of 4 min duration separated by a few minutes of rest, gave significantly lower values than those of the reference method. The reason is the drift in the O_2_ uptake beyond 4 min of exercise. Therefore, the slope calculated from data after 4 min of exercise was less than for the reference method, leading to a calculated accumulated O_2_ deficit 6–15% below the values of the reference method. Method 2 performed similarly to method 1 except for data for one subject. We do not recommend the use of these short-cut methods, at least unless it can be shown that our reference method overestimates the O_2_ demand.

Method 3 with its small steps of increases in exercise intensity every 4 minute is a commonly used protocol for testing. The whole test can be carried out in one session, usually lasting <1 h. The relationship between power and O_2_ demand established by this method was similar to that of the reference method, and consequently the accumulated O_2_ deficit calculated by this method did not differ greatly from that of the reference method. Moreover, since the protocol used is similar to that of finding the lactate threshold, the two tests may be combined. This fast method may serve as a suitable alternative to the reference method. That conclusion is in line with an earlier study on treadmill running (Medbø et al., 1988).

There are non-linear effects at low exercise intensity. For treadmill running, these may be associated with disproportional vertical body movements at low running speeds (see also Medbø et al., 1988). For cycling, they may be related to movements of the legs (repeated accelerations and decelerations) and compensatory use of muscles in the upper body, as addressed above. The effect is more pronounced at high than at low pedaling rates, but it was also present in cycling at only 0.5 Hz. As an extreme measure, we included all measurements below our proposed lower threshold of inclusion in our method 4. The slopes obtained were lower than those of the reference method, but the intercepts were higher. The accumulated O_2_ deficit calculated by this method was 5–6% less than those of the reference method. The mismatch is clearly less if values only slightly below the non-linear threshold are included. Thus, inclusion of a few values slightly below the recommended lower limit does not seem to have much effect. In the results we have only shown this effect for bouts of 10 min duration. There is also a corresponding effect for methods 1–3.

#### Methods Assuming a Fixed, Common Intercept and Few Individual Values

While the slope of O_2_ uptake vs. treadmill speed varies systematically between subjects, the intercept does not vary much (Medbø et al., 1988), see also the outcomes of methods 1 and 2 in this study. This may suggest using one common intercept for all subjects. However, the intercept varies systematically between exercise conditions, as shown in Figure 3 for different pedaling frequencies. This means that a value found for one exercise condition cannot be used for a different condition, as also pointed out earlier (Medbø and Tabata, 1989; Medbø et al., 1999).

Method 5 used a common, fixed intercept taken as the mean for all subjects in the study. That was the recommended alternative to the reference method proposed by Medbø et al. (1988), and that approach has also been recommended by Bickham et al. (2002). That method also worked quite well in this study since the intercepts did not vary greatly between subjects despite considerable variations in the slopes. As pointed out above, the intercept must be established for each exercise condition, which may limit the use of this method.

Method 6 used the gross economy or efficiency principle. Its intercept is zero. The slope was consequently too high, as were the values of the accumulated O_2_ deficit calculated by this method. The outcome of this method also showed the largest between-subjects variability. We do not recommend the use of this method, at least not for cycling and running. As pointed out by Boone and Bourgois (2012), delta efficiency rather than gross efficiency seems to be the best measure of exercise economy.

Method 7 used the net economy or efficiency principle, using resting O_2_ uptake as the intercept. That value of the intercept was clearly too low at only around half the value of the intercepts established by the measured data. The slope established by this method was therefore too high as were the values of the accumulated O_2_ deficit. We do not recommend use of this method, at least not for cycling and running.

Method 8 also used the net economy or efficiency principle, but for this method the intercept was taken as the O_2_ uptake during zero load cycling. That value is too high because of the non-linear effects seen at low exercise intensities addressed above. The slope was therefore lower than that of the reference method, as was the accumulated O_2_ deficit calculated by this method. We do not recommend use of this method, at least not for cycling and running.

We used only one measurement of the steady state O_2_ uptake for methods 5–8. Medbø et al. (1988) recommended using 2–3 measurements as a safeguard since erroneous measurements occasionally occur. In this study we saw only a few poor measurements among around a thousand taken, and we could check those used in the calculations with other values as needed. If that means of validation is not available, we recommend 2–3 measurements rather than just one, as proposed earlier by Medbø et al. (1988).

The use of the gross efficiency principle has been strongly recommended by e.g., Ettema and Lorås (2009), and it has more recently been used by e.g., Andersson and McGawley (2018) to test sprint skiing. If the method is based on measurements of the steady state O_2_ uptake at high powers, it is numerically robust. That holds for all methods using a fixed intercept and few measurements (e.g., methods 5–8 in this study). The zero intercept of method 6 is clearly misleading. Methods 5, 7, and 8 use intercepts that are more realistic. These methods are simple to carry out once the intercept has been established. Consequently, we consider the gross efficiency method to be inferior to any of these alternative methods.

### Suggestions for Further Studies and Development

A basic assumption of the accumulated O_2_ deficit principle is that a linear extrapolation is justified. As pointed out above, at high speeds acceleration of body segments may be a significant part of the work done by the muscles. Since the kinetic energy increases as the square of the speed, there may be significant non-linear effects at high speeds, in particular if the frequency also increases. In line with that, Andersson and McGawley (2018) suggest that non-linear effects may be significant during roller skiing on the treadmill. These facts may suggest non-linear curve fits, but non-linear fits are numerically non-robust and therefore not suited for extrapolations. We here address some possibilities that may deserve further examination. If correctly implemented, the O_2_ demand may be established at the exercise intensity in question, thus eliminating the need for an extrapolation.

#### Short Interval Principle

During short interval training, high intensity exercise is performed for a short time (usually <1 min) before a short rest to recover (usually ≤ 30 s). After the rest period a new effort is made, and this cycle is repeated (see Buchheit and Laursen, 2013, for a review). Because of the short rest periods, there are few variations in the physiological responses to exercise, such as lung ventilation, heart rate and O_2_ uptake, despite large variations in the exercise intensity between exercise and recovery periods. In the Tabata protocol subjects cycle for 20 s, rest for 10 s and continue this 20/10-cycle for 4 min (eight cycles) while the O_2_ uptake is measured continuously (Tabata et al., 1996, 1997). The exercise intensity in those studies was 170% of that corresponding to the maximal O_2_ uptake, which would normally exhaust the subject in <1 min if continued with no break. However, because of the short rest period, subjects are able to continue for ≈4 min before reaching exhaustion.

It is well-established that high lung ventilation is aerobically demanding (Aaron et al., 1992; Dominelli et al., 2014), and consequently more than 10% of the cardiac output may be directed to the respiratory muscles (Harms et al., 1998). As a result, the O_2_ demand of the rest periods is expected to be considerably higher than at quiet rest with no prior high-intensity exercise. If correctly performed, e.g., correcting for high ventilation during the rest periods, a short interval protocol may enable the examination of whether a linear extrapolation is justified for high-intensity exercise, such as treadmill running at high speeds or cross-country skiing on the treadmill, or whether there are significant non-linear effects. It may also allow for direct estimation of the rate of energy release without any extrapolation.

#### Treadmill Exercise at Constant Speed

As pointed out above, accelerations and decelerations of body segments may introduce non-linear effects. If exercise is performed on a treadmill at constant speed, that possible influence may be constant, independent of the physiological demand on the body.

##### Mechanical Assistance

For safety reasons subjects may wear a harness anchored to the ceiling during treadmill exercise. A separate rope attached to the harness may pull the subject forward, thus reducing the need for propulsive force developed by muscles. In that case the O_2_ demand of the exercise is expected to be reduced correspondingly. If the pulling force is recorded and in addition varied between experiments, a relationship between bodily developed propulsion force (or rope pulling force) and O_2_ demand may be established. The proposed design may also allow for measuring the propulsive force needed for treadmill exercise, for example during roller skiing, for comparison with theoretical estimates (Andersson and McGawley, 2018).

##### Varying Treadmill Inclination

Exercise on a level treadmill may be quite easy, while increasing the inclination will increase the physiological demand at constant speed. A relationship between treadmill inclination and steady state O_2_ uptake may be established for e.g., cycling on the treadmill or roller skiing and used for extrapolations if a linear relationship can be found. Our unpublished findings suggest that for running a corresponding relationship is not linear. The reason for the latter observation may be related to a vertical movement during the stride that varies with the inclination.

## Conclusions

Relationships between exercise intensity and O_2_ demand vary between persons and should therefore be established individually. The computational method used for calculating the accumulated O_2_ deficit has considerable influence on the outcome. A protocol using 4 min stepwise increases in exercise intensity may enable the establishment of reliable relationships within 1 h of testing. That approach may be a suitable alternative to the reference method. Commonly used methods based on a fixed *y*-intercept (gross efficiency or net efficiency) are not recommended. Further methodological studies are needed, as proposed above.

## Data Availability Statement

The raw data supporting the conclusions of this article will be made available by the authors, without undue reservation.

## Ethics Statement

Ethical review and approval was not required for the study on human participants in accordance with the local legislation and institutional requirements. The patients/participants provided their written informed consent to participate in this study.

## Author Contributions

JIM and BW developed the main idea of the study together. JIM conducted the experiments, analyzed the data, and drafted a manuscript. JIM and BW jointly revised the manuscript and approved the final version.

## Conflict of Interest

The authors declare that the research was conducted in the absence of any commercial or financial relationships that could be construed as a potential conflict of interest.

## Publisher's Note

All claims expressed in this article are solely those of the authors and do not necessarily represent those of their affiliated organizations, or those of the publisher, the editors and the reviewers. Any product that may be evaluated in this article, or claim that may be made by its manufacturer, is not guaranteed or endorsed by the publisher.
